# High risk score of breast cancer by artificial intelligence (AI) on screening mammograms: a review of negative and cancer cases

**DOI:** 10.1007/s00330-026-12579-4

**Published:** 2026-05-06

**Authors:** Marit A. Martiniussen, Marie B. Bergan, Merete U. Kristiansen, Nataliia Moshina, Anne Sofie F. Larsen, Marthe Larsen, Fredrik A. Dahl, Solveig Hofvind

**Affiliations:** 1 Department of Radiology and Nuclear medicine, Østfold Hospital Trust, Kalnes, Norway; 2 University of Oslo, Institute of Clinical Medicine, Oslo, Norway; 3 Department of Breast Cancer Screening, Cancer Registry, Norwegian Institute of Public Health, Oslo, Norway; 4 Norwegian Computing Center, Oslo, Norway; 5 Department of Health and Care Sciences, UiT, The Artic University of Norway, Tromsø, Norway

**Keywords:** Breast neoplasms, Mammography, Mass screening, Artificial intelligence

## Abstract

**Objectives:**

To investigate mammographic features associated with high artificial intelligence (AI) risk scores as provided by two AI models applied to screening mammograms.

**Materials and methods:**

This retrospective study included 130,031 screening mammograms from 42,371 women attending BreastScreen Norway, 2008–2018. Two AI models (A and B) developed for cancer detection on screening mammograms were applied. An informed radiological review was conducted for mammograms within the highest 5% of AI risk scores by both models in two study samples: (1) High AI risk score, but no breast cancer detected within 6 years (*n* = 120), and (2) High AI risk score in mammograms with screen-detected cancers (*n* = 120). Mammographic density (BI-RADS a–d), features (mass, spiculated mass, asymmetry, architectural distortion, calcification alone, and density with calcification), and radiologists’ interpretation scores (1–5) were analyzed descriptively.

**Results:**

Mammographic density was higher in sample 1 compared to sample 2 (BI-RADS d: 11% vs 3%, respectively). In sample 1, calcifications alone were the most frequent AI-marked feature (model A: 72%; model B: 68%), predominantly with amorphous morphology and a cluster distribution, and 76% were interpreted as benign by the radiologists (interpretation score 1). In sample 2, a spiculated mass was the most frequent mammographic feature among the screen-detected cancers (29%).

**Conclusion:**

Mammograms assigned high AI risk scores exhibit distinct features depending on screening outcome. Systematic characterization of these features may help refine AI thresholds, improve specificity, reduce AI false-positive findings, and decrease the recall rate in breast cancer screening.

**Key Points:**

***Question*** *Knowledge about mammographic features associated with high AI risk scores is essential for distinguishing cancer from non-cancer cases.*

***Findings*** *Calcifications were the dominant feature in non-cancers in screening mammograms with high AI risk score, whereas spiculated mass was the most frequent feature among cancers.*

***Clinical relevance*** *Calcifications in non-cancer screening mammograms with a high AI risk score were frequently interpreted as benign or probably benign by radiologists. This knowledge may help refine AI thresholds and thereby improve specificity and reduce false-positive results in mammographic screening.*

**Graphical Abstract:**

**Figure:**
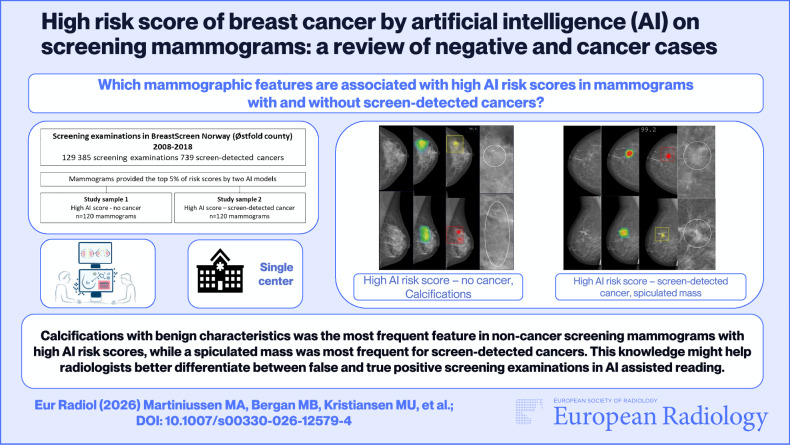

## Introduction

Breast cancer is the most common cancer and the leading cause of cancer-related death among women worldwide, with 2.3 million new cases and nearly 666,000 deaths in 2022 [1]. Mammographic screening is recommended by international health organizations to facilitate early detection [2, 3] and has been shown to reduce the incidence of advanced disease, treatment burden, and breast cancer-specific mortality [4, 5]. Nevertheless, screening entails potential harm, including overdiagnosis and false-positive screening results [5]. Overdiagnosis is the detection of breast cancers that would not have become clinically apparent in the women’s lifetime in the absence of screening [4] and may lead to overtreatment. False-positive screening results may induce psychological distress and anxiety and are associated with decreased adherence to subsequent screening rounds [6, 7], despite these women exhibiting an increased long-term risk of breast cancer [8]. Moreover, recall assessments are resource-intensive, highlighting the need for optimized recall rates to ensure sustainable screening programs [9].

Interpreting mammograms is inherently challenging, as some imaging features of negative cases may mimic cancer while others with subtle appearances can represent true cancers, underscoring the assessment and diagnostic complexity [10]. Accurate characterization of mammographic features is therefore critical in clinical practice [11]. The Breast Imaging and Data System (BI-RADS) provides a standardized lexicon and assessment framework that supports consistent interpretation and communication of malignancy likelihood and has been widely adopted by breast radiologists worldwide [12].

Artificial intelligence (AI) models based on deep learning have demonstrated promising performance on mammograms and breast cancer detection, with the potential to reduce recall and radiologists’ screen-reading volume [13–15]. However, fundamental questions remain regarding the specific types of lesions these models detect or miss. Evidence comparing mammographic features associated with high AI risk scores in women with and without cancer remains limited [15–17]. Elucidating how AI models respond to distinct mammographic features may help identify patterns associated with AI risk scores on cases with and without breast cancer. Such knowledge could improve interpretive accuracy, improve workflow efficiency, and foster radiologists’ trust and acceptance of AI-assisted screening.

In this study, we aimed to characterize and compare mammographic features in screening examinations with high AI risk scores for women with and without screen-detected breast cancer, in order to better understand the specific features identified as suspicious by two AI models.

## Materials and methods

This retrospective study has legal basis in accordance with Articles 6 (1) (e) and 9 (2) (j) of the GDPR and was approved by the Regional Committees for Medical and Health Research Ethics (REC#13294, REC#11022). The data was disclosed with legal basis in the Personal Health Data Filing system Act Section 19a-h [18] and the Cancer Registry Regulation Sections 3–1 [19].

### BreastScreen Norway

BreastScreen Norway, administered by the Cancer Registry, Norwegian Institute of Public Health, invites about 680,000 women aged 50–69 for biennial two-view digital mammography (craniocaudal, CC, and mediolateral oblique, MLO, views) screening [20]. Two radiologists independently score the screening mammograms from 1 to 5, where 1 indicates normal findings; 2, probably benign; 3, intermediate suspicion; 4, probably malignant; and 5, high suspicion of malignancy. If either or both radiologists assign a score of ≥ 2, a consensus meeting will determine whether to recall the woman for further assessment. In 2017–2021, attendance was 76%, recall 3.3%, screen-detected cancer 6.2 per 1000 and interval cancer 1.8 per 1000 screening examinations [20].

### Study sample 1 and 2

This retrospective study included 130,031 digital screening mammograms from 42,371 women screened at Østfold Hospital between January 2, 2008, and December 19, 2018. All women were screened using MAMMOMAT Inspiration (Siemens Healthcare), and pseudonymized mammograms were retrospectively analyzed by AI models A and B.

**Fig. 1:**
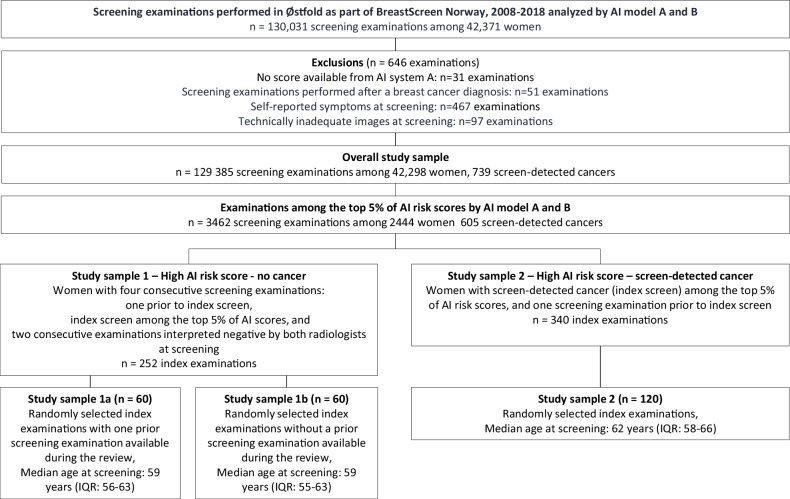
Flowchart of study samples 1 and 2

After excluding examinations with missing AI risk scores, those performed after breast cancer diagnosis, those associated with self-reported symptoms at screening and with technically inadequate images, 129,385 screening examinations remained, including 739 screen-detected cancers and 208 interval cancers (Fig. 1).

Inclusion criteria for study sample 1 were: a screening examination with high AI risk score (top 5%) and no cancer diagnosis, interpreted negative (score 1 + 1) by both the radiologists at screening (defined as index screen); confirmation of no cancer through two subsequent negative screening rounds, (up to 6-years follow-up for interval cancers); and a screening examination prior to index screen to enable temporal comparison, resulting in a set of four consecutive screening examinations. The examinations were divided into study sample 1a, with prior mammograms, and study sample 1b, without prior mammograms, used in a review performed by two radiologists (Fig. 1).

Study sample 2 was derived from 739 screen-detected cancer cases. The inclusion criteria were an AI risk score within the highest 5% by both AI models and at least one prior screening examination (Fig. 1).

### AI model A and B

**Fig. 2:**
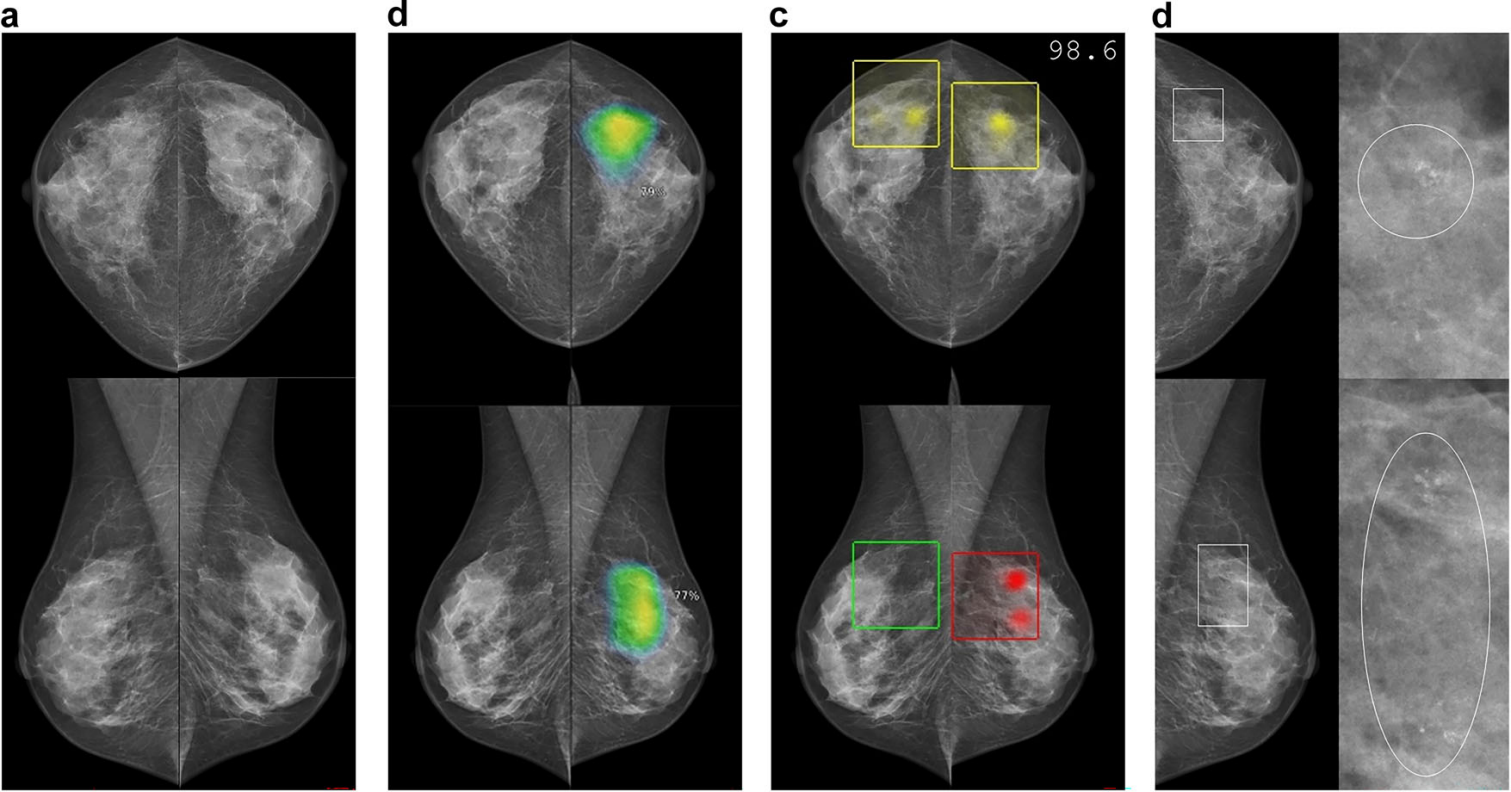
Screening mammograms in craniocaudal (CC) and mediolateral oblique (MLO) views as they appeared in a clinical setting (**a**), with risk scores and markings by AI model A (**b**) and B (**c**) and magnification of benign small clusters of amorphous calcifications (**d**)

AI model A (Lunit INSIGHT MMG version 1.1.7.2, Conformité Européenne-marked (CE-marked) and Food and Drug Administration (FDA) cleared) provided a continuous breast level malignancy risk score, from 0 to 100% and heatmap markings on the mammograms for risk scores ≥ 10% (Fig. 2a, b) [21].

AI model B was an in-house model from the Norwegian Computing Center (NCC), developed as a research project in collaboration with BreastScreen Norway. The model is described elsewhere [22]. The mammograms from Østfold were not accessed in any way during the training and cross-validation of the model. The model assigned risk scores on a continuous scale from 0 to 100, highlighting a square region of interest in each view, colored green, yellow, orange or red with increasing risk of cancer (Fig. 2c). The model provided one or more markings within all square regions of interest but the green ones. Overall performance for these two models for an almost identical study population has been published [23].

### Variables of interest

Screen-detected cancer was histologically verified as ductal carcinoma in situ (DCIS) or invasive breast cancer diagnosed after further assessment due to mammographic findings. Examinations with high AI risk scores were defined as those within the highest 5% of AI risk scores. AI false positives were examinations with a high AI risk score but no cancer (Study sample 1).

Mammographic density was categorized according to BI-RADS 5th edition, a–d [12] by the radiologists, in the consensus review. Mammographic features were classified using a modified BI-RADS lexicon: a mass, a spiculated mass, asymmetry, architectural distortion, calcifications alone and density with calcifications [24]. Calcifications were further described by morphology (coarse heterogeneous, amorphous, fine pleomorphic and fine linear or branched calcifications) and by distribution (diffuse, regional, clustered, linear and segmental distribution, isolated/not clustered) [12]. Mammograms with calcifications alone or with density were considered one group in the calculations. Clustered calcifications were described according to size, either < 5 mm or ≥ 5 mm.

### Radiological review

Two experienced breast radiologists (M.U.K. and M.A.M., with 21 and 8 years of experience in screen reading, respectively) performed a consensus-based informed review of screening mammograms with AI-marked regions. The two study samples were reviewed separately, with reviewers aware of the presence or absence of cancer.

For cases in study sample 1, mammographic density, mammographic feature associated with the AI-marked location and radiologists’ interpretation score (1–5) were registered during the review. For cases in study sample 2, mammographic density, mammographic feature of the screen-detected cancer and the location of AI markings were registered. The location of the AI markings was compared with the true cancer location confirmed by diagnostic mammography.

### Statistical analysis

Frequencies and percentages were reported for mammographic breast density, AI-marked mammographic features, correct location of the AI marking and radiological interpretation scores. Group differences were assessed using chi-square (χ²) test, with a significance level of 0.01. Analyses were performed using Stata (StataCorp. 2025. Stata Statistical Software: Release 19).

## Results

### Study sample 1 and 2

Of the 252 examinations that met the inclusion criteria for both AI models in study sample 1, 120 were randomly selected for radiological review: study sample 1a (*n* = 60) with prior mammograms available for comparison during the review, and study sample 1b (*n* = 60) without prior mammograms available. Of the 340 screening mammograms included in study sample 2, 120 were randomly selected for radiological review. Mean age in study sample 1 was 59 years (IQR 56–63 for study sample 1a, IQR 56–63 for study sample 1b) and 62 years (IQR 58–66) for study sample 2 (Fig. 1).

### Mammographic density

Mammographic density BI-RADS category b was the most common classification in both study samples, accounting for 46.7% (56/120) of the examinations in study sample 1 (high AI risk score, without cancer) and 55.0% (66/120) in study sample 2. BI-RADS category d was observed in 10.8% (13/120) of the cases in study sample 1, and 3.3% (4/120) in study sample 2 (Table 1).

**Table 1:** The distribution of BI-RADS (5th edition) mammographic density (a–d) in study sample 1 (mammograms within the 5% highest of AI risk scores, without cancer) and study sample 2 (mammograms with screen-detected cancers within the 5% highest of AI risk scores)

BI-RADS 5th ed	Study sample 1			Study sample 2	Study sample 1 versus 2
Mammographic density	Study sample 1a^α^ with priors *n* = 60	Study sample 1b^β^ without priors *n* = 60	Study Sample 1 *n* = 120	*n* = 120	*p*-value*
	*n* (%)	*n* (%)	*n* (%)	*n* (%)	
a	5 (8.3%)	11 (18.3%)	16 (13.3%)	28 (23.3%)	0.004
b	31 (51.7%)	25 (41.7%)	56 (46.7%)	66 (55.0%)	
c	16 (26.7%)	19 (31.7%)	35 (29.2%)	22 (18.3%)	
d	8 (13.3%)	5 (8.3%)	13 (10.8%)	4 (3.3%)	

^α^ Screening mammograms with top 5% of AI risk scores by model A and B, but without cancer, reviewed by radiologists with prior mammograms available for comparison

^β^ Screening mammograms with the top 5% of AI risk scores by model A and B, but without cancer, reviewed by radiologists without prior mammograms available for comparison

* Overall *p*-value from chi-squared (χ²) test for association between mammograms without and with cancer and mammographic density

### Location of AI markings

In the radiological review of AI-marked lesions, both AI models correctly localized all but one screen-detected cancer in at least one view, with the same case mislocalized by both AI models. Model A correctly localized the cancer in both views in 95.8% of cases (115/120), whereas model B did so in 98.3% (118/120) (Table 2).

**Table 2:** Distribution of correct and incorrect location of artificial intelligence (AI) markings on cancer lesions by view (craniocaudal, CC and mediolateral oblique, MLO) for AI models A and B in study sample 2

AI markings by view	Study sample 2	
	AI model A *n* = 120	AI model B *n* = 120
	*n* (%)	*n* (%)
Correct location in CC and MLO views	115 (95.8%)	118 (98.4%)
Correct location in CC view only	4 (3.4%)	0 (-)
Correct location in MLO view only	0 (-)	1 (0.8%)
Incorrect location	1 (0.8%)	1 (0.8%)

### Mammographic features

**Fig. 3:**
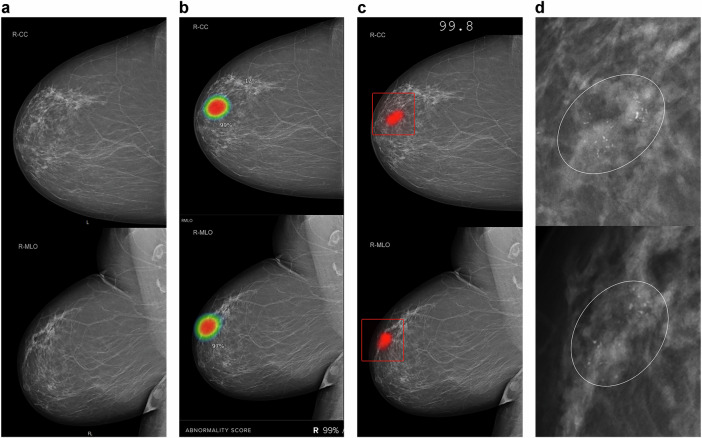
Screening mammogram of the right breast in craniocaudal (CC, upper panels) and mediolateral oblique (MLO, lower panels) views with a screen-detected cancer (**a**). Both model A (**b**) and B (**c**) provided high AI risk scores (top 5%) and marked the correct cancer location in both views. The radiologists classified the cancer as calcification with fine pleomorphic morphology with regional distribution (magnitude) (**d**)

**Fig. 4:**
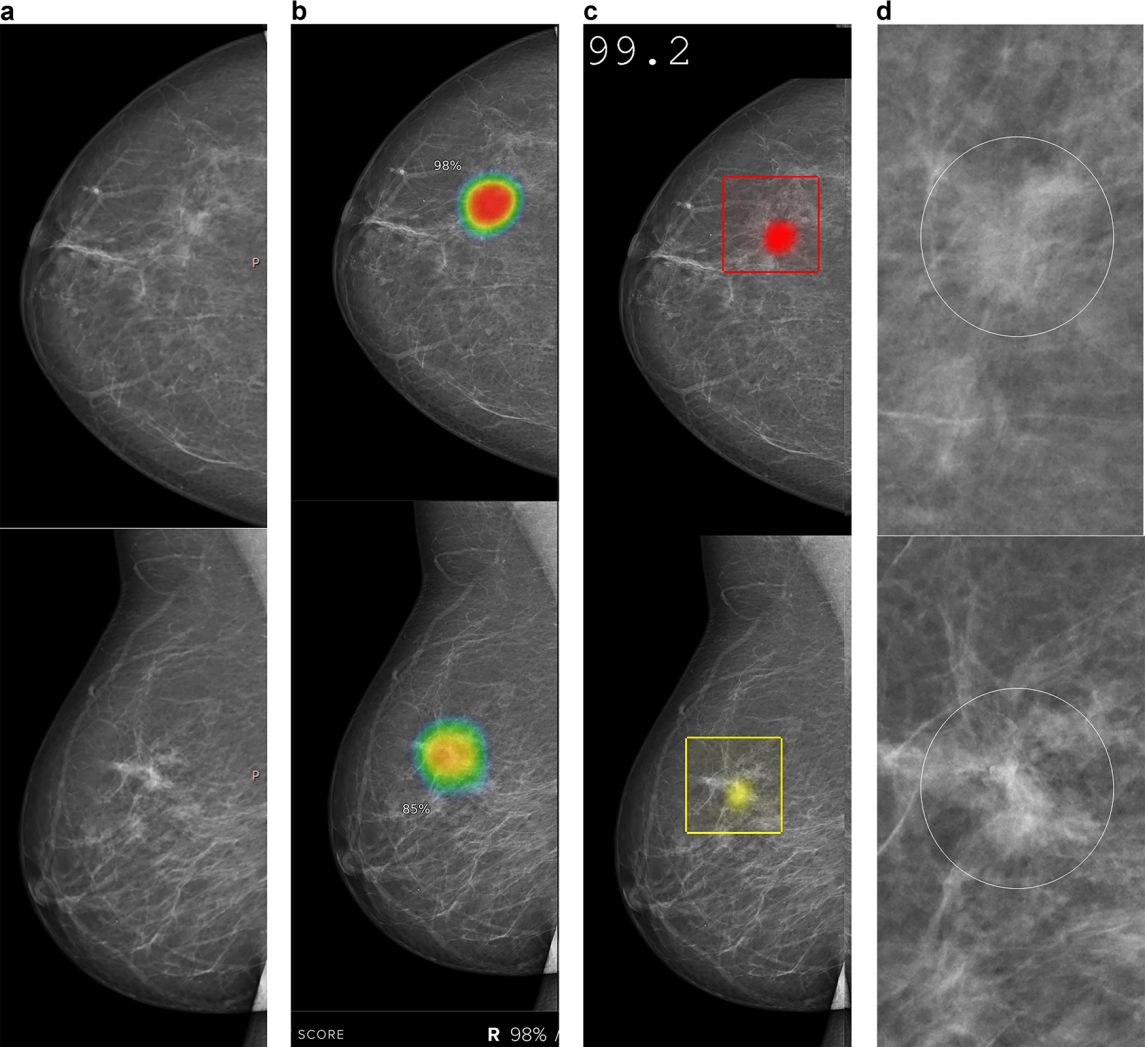
Screening mammogram of the right breast, including craniocaudal (CC, upper panels) and mediolateral oblique (MLO, lower panels) views of a female with a screen-detected cancer (**a**). AI model A (**b**) and B (**c**) provided a high risk score (top 5%) and correctly located the cancer in both views. Magnitude of the location marked by both models, classified as a spiculated mass by the radiologists (**d**)

In study sample 1, the most frequent AI-marked mammographic feature was calcifications alone, identified in 71.7% (86/120) of the cases by model A and 67.5% (81/120) by model B. In study sample 2, calcifications alone were marked by AI in 25.0% (30/120) of the cancer cases (Fig. 3). The two most frequent features in study sample 2 were a spiculated mass (Fig. 4), observed in 29.2% (35/120), and a mass in 26.7% (32/120) of the cancer cases. A spiculated mass was not observed in study sample 1 (Table 3). The mammographic features marked in study sample 1 and at the cancer location in study sample 2 differed statistically (*p* < 0.001).

**Table 3:** Distribution of mammographic features classified retrospectively by radiologists, associated with AI markings by model A and B in study sample 1 (mammograms with the highest 5% of AI risk scores, without cancer) and at the cancer location in study sample 2 (screen-detected cancers within the highest 5% of AI risk scores)

Mammographic features	Study sample 1		Study sample 2
	AI model A (*n* = 120)	AI model B (*n* = 120)	Cancer location (*n* = 120)
	*n* (%)	*n* (%)	*n* (%)
Mass	15 (12.5%)	17 (14.2%)	32 (26.7%)
Spiculated mass	0 (-)	0 (-)	35 (29.2%)
Asymmetry	13 (10.8%)	14 (11.7%)	2 (1.7%)
Architectural distortion	3 (2.5%)	4 (3.3%)	11 (9.2%)
Calcifications alone	86 (71.7%)	81 (67.5%)	30 (25.0%)
Density with calcifications	2 (1.7%)	3 (2.5%)	10 (8.3%)
Negative	1 (0.8%)	1 (0.8%)	0 (-)

In study sample 1, the most frequent calcification morphology was amorphous, marked in 76.1% (67/88) of the examinations for model A and 77.4.0% (65/84) for model B, while in study sample 2, it was 25% (10/40). The most frequent morphology in study sample 2 was fine pleomorphic, present in 37.5% (15/40) of the cancer cases, while in study sample 1, it was 1.1% (1/88) marked by model A and 1.2% (1/84) by model B. The most frequent calcification distribution in study sample 1 was clusters, found in 48.9% (43/88) of the calcifications marked by model A and 50.0% (42/84) marked by model B (Fig. 2d). These clusters were evenly distributed in < 5 mm and ≥ 5 mm for both models. Diffuse distribution was observed in 26.1% (23/88) of the cases marked by model A and 25.0% (21/84) by model B in study sample 1, while that distribution was not observed in any of the cancer cases in study sample 2 (Table 4). The morphology and distribution of the calcification differed statistically between study samples 1 and 2 (*p* < 0.001).

**Table 4:** Distribution of calcification characterization associated with AI markings by model A and B in study sample 1 (mammograms with the highest 5% of AI risk scores, without cancer) and for the cancer cases in study sample 2 (screen-detected cancers with the highest 5% of AI risk scores), using the number of cases with calcification alone and density with calcifications for each study sample as the denominator

Calcification characterization	Study sample 1		Study sample 2
	AI model A (*n* = 88)	AI model B (*n* = 84)	AI model A and B (*n* = 40)
Morphology	*n* (%)	*n* (%)	*n* (%)
Benign	14 (15.9%)	12 (14.3%)	0 (-)
Amorphous	67 (76.1%)	65 (77.4%)	10 (25.0%)
Coarse heterogeneous	6 (6.8%)	6 (7.1%)	8 (20.0%)
Fine pleomorphic	1 (1.1%)	1 (1.2%)	15 (37.5%)
Fine linear/fine linear branching	0 (-)	0 (-)	7 (17.5%)
Distribution			
Diffuse	23 (26.1%)	21 (25.0%)	0 (-)
Regional	15 (17.0%)	13 (15.5%)	13 (32.5%)
Clustered, < 5 mm	22 (25.0%)	21 (25.0%)	4 (10.0%)
Clustered, ≥ 5 mm	21 (23.9%)	21 (25.0%)	14 (35.0%)
Segmental	6 (6.8%)	6 (7.1%)	6 (15.0%)
Linear	0 (-)	0 (-)	3 (7.5%)
Isolated/not clustered	1 (1.1%)	2 (2.4%)	0

### Radiological interpretation score

In study sample 1a, the radiologists assigned an interpretation score of 1 (negative) to 93.3% (56/60) and a score of 2 (probably benign) to 6.7% (4/60) of the examinations, where prior mammograms were available for comparison. In study sample 1b, where no prior mammograms were available, 58.3% (35/60) were assigned a score of 1, 40.0% (24/60) a score of 2, and 1.7% (1/60) a score of 3 (intermediate suspicion) (Table 5).

**Table 5:** Distribution of a modified BI-RADS interpretation score assigned by the radiologists at the informed consensus review of study sample 1 (mammograms with the highest 5% of AI risk scores, without cancer) for study sample 1a (prior mammograms available for comparison at the review) and study sample 1b (no prior mammograms available for comparison at the review)

Radiologists’ interpretation score	Study sample 1a *n* = 60	Study sample 1b *n* = 60	Study sample 1a versus 1b
	*n* (%)	*n* (%)	*p*-value*
1	56 (93.3%)	35 (58.3%)	< 0.001
2	4 (6.7%)	24 (40.0%)	
3	0 (-)	1 (1.7%)	
4–5	0 (-)	0 (-)	

* Associations between study samples 1a and 1b with the interpretation score by the radiologists

## Discussion

In this retrospective review of screening mammograms with high AI risk scores, calcifications were the predominant finding in cases without cancer, whereas a spiculated mass was the most frequent feature in mammograms with screen-detected cancers. The findings highlight distinct patterns associated with false-positive and true-positive AI risk scores.

Examinations with high AI risk scores without cancer were more frequently classified with high mammographic density than those with high scores and cancers, suggesting that AI performs less accurately in dense breasts. This aligns with Nguyen et al, who reported higher odds of a false-positive AI risk score in dense versus less dense breasts [25]. Dense and overlapping breast tissue might mask cancers or mimic suspicious findings without representing a malignancy, underscoring the interpretation complexity for radiologists and AI models [26]. High mammographic breast density is associated with increased risk of developing breast cancer, as well as a reduced recurrence-free survival compared to low mammographic density [27]. Consequently, understanding AI performance in mammographic dense breasts is essential for future discussion of personalized risk-based screening for breast cancer.

At a threshold that defines AI positive mammograms as those falling within the highest 5% of AI risk scores, slightly less than 90% of AI positive mammograms do not represent cancer. Knowledge of which mammographic features AI models identify as suspicious may help radiologists select cases that truly represent breast cancer and avoid work-up with negative outcomes, thereby increasing the specificity. Spiculated masses were the most frequent mammographic feature in study sample 2 (screen-detected cancers with high AI risk scores), and absent in study sample 1 (high AI risk score, without cancer), underlining a clear distinction between AI true positives and AI false positives. Additionally, masses and density with calcifications were more frequent in study sample 2 compared to study sample 1, indicating these features represent more suspicious mammographic findings. These observations are consistent with descriptors outlined in the BI-RADS 5th edition. In study sample 1, the most frequent AI-marked mammographic feature was calcifications alone, about 70%, while this feature was present in about 30% of the cases in study sample 2. Calcifications are common in women with and without breast cancer [28], but depending on their morphology, they might indicate DCIS, invasive cancer or benign lesions [29]. The morphological characteristics of the calcifications differed between the two study samples. In study sample 1, amorphous calcifications in small clusters of < 5 mm were most frequent, while fine pleomorphic calcifications in clusters of ≥ 5 mm were most frequent in study sample 2. These patterns align with a retrospective study of calcifications on mammograms with and without cancers [30] and the BI-RADS 5th edition descriptions suggesting that fine pleomorphic calcifications likely represent malignancy, while multiple clustered amorphous calcifications are likely benign [12].

Although clusters of calcifications are recognized as an independent risk factor for breast cancer, most mammograms with such clusters do not include malignancy [29]. Notably, cancers presenting primarily as calcifications tend to have poorer survival than those presenting as a spiculated mass [24], emphasizing the importance of early detection and accurate interpretation of calcification patterns.

Among the AI false positives, typical benign calcifications, such as vascular, skin, coarse and dystrophic types, were relatively infrequent, about 15% in study sample 1. Previous similar studies report benign calcifications as the most frequent type in AI false positives [16, 31]. Our analyses included only mammograms with a high AI risk score by both AI models, and results might differ if cases flagged by a single model were considered, highlighting how AI interpretation can vary depending on model and thresholds. In addition, radiologists performing the reviews might have different perceptions of the definitions of benign features.

Further, knowledge about the different models’ performance in terms of breast density and mammographic features is important for defining thresholds for negative and positive cases. Validation studies will also be important after the implementation of AI-assisted mammographic screening, and possibly, automated validation of AI models will be part of a standardized quality assurance in the future.

During the radiological review, mammograms in study sample 1 were assigned interpretation scores by the radiologists. As no cancers were present, most cases received a score of 1 (normal) or 2 (probably benign). However, both radiologists had given a score of 1 on both breast on the index screen and two subsequent screening rounds, underscoring the challenge related to automation bias [32]. Access of prior mammograms yielded lower scores, emphasizing the value of temporal comparison. The AI models used in this study lacked this capability, but integrating temporal analysis in the future may enhance specificity and reduce recall rates.

A major strength of this study was its use of data from a regular screening setting, providing clinically relevant insight. Few studies have examined mammographic features driving AI false positive [16, 31], and understanding these features might help radiologists reduce the recall rate while informing future AI development. Limitations included the single-center study design and the subjective nature of the radiological review [33]. Radiologists were aware that study sample 1 included only non-cancer mammograms and study sample 2 only screen-detected cancers. This may have influenced the radiologists’ interpretation scores of study sample 1. It may also have affected mammographic feature classification in study sample 2, where some cancers could be misinterpreted as negatives. However, in this study, it was interesting to characterize different mammographic features of cancers, even subtle signs. Due to the limited number of cases presenting with both masses and calcifications, these were combined with calcifications alone into a single analytical group, which should be considered a limitation. Further, no information about the underlying causes of calcifications in study sample 1, including prior surgery for benign lesions or breast reduction, was available.

In conclusion, mammograms with high AI risk scores display distinct features depending on the presence of cancer. Calcifications alone, typically assessed as low suspicion by radiologists, were the predominant finding in AI false-positive cases. Understanding these patterns provides valuable insights into AI-assisted screen-reading and may support strategies to reduce the recall rate. Furthermore, these findings can facilitate the development of more refined AI models and contribute to the future implementation of personalized, risk-based breast cancer screening.

## Abbreviations

AI: Artificial intelligence
